# Editorial: Youth and Winter Sports

**DOI:** 10.3389/fspor.2021.696232

**Published:** 2021-06-02

**Authors:** Grégoire P. Millet, Fabien Ohl

**Affiliations:** Institute of Sport Sciences, University of Lausanne, Lausanne, Switzerland

**Keywords:** winter sports, physiology, biomechanics, congress, Olympic, youth

## Congress “Youth and Winter Sports”

Just before the beginning of the COVID-19 pandemic, Lausanne—known as the “Olympic capital” since it is the home of the International Olympic Committee—had the privilege and opportunity to organise the last international sporting event not affected by the virus. The 2020 Winter Youth Olympic Games was the third edition of the Winter Youth Olympic Games (YOG) and was held in Lausanne between January 9 and 22, 2020. The event featured 8 sports and 16 disciplines, including ski mountaineering and women's Nordic combined for the first time at the Olympics. A total of 1,788 athletes from 79 nations participated. Among several novelties was the location of the “Olympic village” on the campus of the University of Lausanne. The YOG was an opportunity to bring the academic and sports worlds closer together. The University of Lausanne collaborated with the IOC on various educational and scientific programmes for athletes and the public. Thus, in collaboration with IOC's Olympic Studies Centre and several academic institutions, the Institute of Sport Sciences (ISSUL) organised the congress “*Youth and winter sports*” https://wp.unil.ch/cyws20/program/ on January 7 and 8. It aimed to bring together sports scientists, coaches, and medics with an interest in winter sports, and around 200 people attended the congress.

Several points (not only the pre-COVID date!) made this congress very special:

1. Free access for all UNIL students and all athletes or staff registered for the YOG with the goal of fruitful exchanges between scientists and practitioners. The Olympic village was only 200 m from the congress location, and we were visited by elite coaches.
2. This congress was officially a component of the educational program of the YOG overviewed by UNIL.
3. This congress was multidisciplinary with key notes and parallel sections on both life sciences and social sciences. We received communications about physical preparation, physiology, biomechanics, sports medicine, rehabilitation, history, sociology, management, psychology, and teaching.
  The following world-class experts gave six key-note presentations:
  - Milena Parent (Canada)—Governance and legacy of the YOG
  - Hans-Christer Holmberg (Sweden)—Biomechanics and physiology in nordic skiing
  - Andrew Denning (USA)—History of the human–environment relationships in the Alps through the sport of skiing
  - Erich E. Muller (Austria)—Biomechanics and prevention of injuries in alpine Skiing
  - Oyvind Sandbakk (Norway)—Norwegian success in winter sports
  - Laurent Schmitt (France) – Martin Fourcade's physiological adaptations-−10 years of training and monitoring.
4. This congress was not supported by a scientific society and was a one-shot organisation connected to the YOG.
5. The congress was supported by Frontiers in Sport and Active Living with the possibility for scientists to submit articles to the present Research Topic.

## Research Topic “Youth and Winter Sports”

Though the Research Topic is multidisciplinary, which is in line with the content of the congress, only articles concerning exercise physiology, biomechanics, and nutrition have been published.

Four articles focused on biomechanical considerations in alpine skiing:

1. Pavailler et al. assessed the differences in glide time, ski edging, and plantar pressure distribution in national and regional cross-country skiers. The elite skiers exhibited better techniques, which is evidenced by the higher relative glide time induced by a larger body mass transfer above the ski particularly at the beginning of the gliding phase.
2. Nimmervoll, Çakmak et al. investigated the forces within the Alpine ski boots and reported that the location of the sensors is paramount for relevant data collection: the insole sensors located in the heel and front areas combined with the shaft sensors provide reliable data on forward and backward leanings.
3. Nimmervoll, Eckerstorfer et al. displayed a new method to release ski binding with an industrial robot. This method allows for the analysis of the respective forces and the release-retention behaviour of ski bindings.
4. Delhaye et al. investigated the kinetic and kinematic specificities of different line strategies (i.e., Z long straight line and sharper turns vs. S short straight line and long curved turns) in Giant slalom and the effects on the alpine skier performance and energy dissipation. Overall, these two strategies lead to similar energy dissipation characteristics, and the authors concluded that elite skiers need to possess a variety of these strategies.

Two articles assessed the talent development in youth cross-country skiers:

1. Zoppirolli et al. reported the changes in anthropometric and physiological characteristics of male and female young cross-country skiers. They showed that, during the late teenage period, high values and threshold levels of VO_2max_ appeared to be good indicators of further talent.
2. Wehrlin and Steiner similarly reported that early (16 and 19 years of age) physiological characteristics (i.e., high total haemoglobin mass expressed in g/kg) are relevant predictors of success at the senior level in endurance sports like cross-country skiing or triathlons.

Finally, this Research Topic includes three reviews (compression garments, injuries, and nutritional considerations) relevant to all winter sports:

1. Yang et al. reviewed 18 studies about the effects of compression garments on performance, drag, and vibrations in several winter sports.
2. Xu et al. reviewed 39 studies on injury factors in winter sports and provided much interesting information: The highest injury incidence was recorded in freestyle skiing, followed by alpine skiing and snowboarding. The most injured body parts were the knees (30%), head and face (12%), shoulders and collarbone (10%), and lower back (9%). The most common injury types were joint and ligament injury (41%), fracture and bone stress (24%), concussion (11%), and muscle/tendon injury (11%).
3. Hannon et al. provided an outstanding guideline on nutrition in youth winter sports athletes. Special considerations related to the environmental conditions as well as the maturation process in these athletes have been discussed, and energy, vitamin D, calcium, iron, and hydration have been analysed.

## Author Contributions

All authors listed have made a substantial, direct and intellectual contribution to the work, and approved it for publication.

## Conflict of Interest

The authors declare that the research was conducted in the absence of any commercial or financial relationships that could be construed as a potential conflict of interest.

